# Draft Genome Sequence of a Quorum-Sensing Bacterium, Dickeya sp. Strain 2B12, Isolated from a Freshwater Lake

**DOI:** 10.1128/genomeA.01542-14

**Published:** 2015-02-05

**Authors:** Kian-Hin Tan, Kit-Yeng Sheng, Chien-Yi Chang, Wai-Fong Yin, Kok-Gan Chan

**Affiliations:** a Division of Genetics and Molecular Biology, Institute of Biological Sciences, Faculty of Science, University of Malaya, Kuala Lumpur, Malaysia; b Interdisciplinary Computing and Complex BioSystems (ICOS) research group, School of Computing Science, Newcastle University, Newcastle upon Tyne, United Kingdom; c The Centre for Bacterial Cell Biology, Medical School, Newcastle University, Newcastle upon Tyne, United Kingdom

## Abstract

Dickeya sp. strain 2B12 was isolated from a freshwater lake in Malaysia. Here, we report the draft genome sequence of Dickeya sp. 2B12 sequenced by the Illumina MiSeq platform. With the genome sequence available, this genome sequence will be useful for the study of quorum-sensing activity in this isolate.

## GENOME ANNOUNCEMENT

All members of the Enterobacteriaceae family that are pathogenic to plants, both pectolytic (Erwinia carotovora and Erwinia chrysanthemi) and nonpectolytic (Erwinia amylovora), were assigned into a new genus, Erwinia, in 1917 (1). Despite the original notion that Erwinia chrysanthemi is a pathogen of chrysanthemum (2), and thus named as such, it was later discovered to infect a wide range of plant hosts (3, 4). E. chrysanthemi strains were classified into several pathovars according to pathogenicity on host plants and biochemical and physiological differences (5). This species was later transferred into a new genus called Dickeya, which now comprises eight species (D. chrysanthemi, D. dadantii, D. dianthicola, D. dieffenbachiae, D. zeae, D. paradisiaca, D. solani, and D. aquatica) (1, 2, 6). Here, we present a draft genome sequence of Dickeya sp. strain 2B12, isolated from a fresh lake water sample.

The genome of Dickeya sp. 2B12 was sequenced by an Illumina MiSeq sequencer with a 150-bp read chemistry. In brief, genomic DNA of Dickeya sp. 2B12 was extracted from an overnight culture using a Master Pure DNA purification kit (Epicentre, Inc., Madison, WI, USA). The quality and quantity of the DNA was assessed via a NanoDrop spectrophotometer (ThermoScientific, Waltham, MA, USA) and a Qubit 2.0 fluorometer (Life Technologies, Carlsbad, CA, USA). Then, 50 ng of DNA was used to construct a sequencing library using a Nextera DNA sample prep kit (Illumina, San Diego, CA, USA) according to the manufacturer’s protocol. The library was quantified by a Qubit 2.0 fluorometer and Bioanalyzer high-sensitivity chip (Agilent Technologies, Santa Clara, CA, USA). A sequencing run was set up to generate 2 × 150 base-paired reads.

The raw reads were trimmed at Q30, resulting in 1,745,868 reads with an average length of 144.3 bp. The reads were assembled *de novo* using CLC Genomic Workbench 6 (CLC Bio, Denmark), giving 120 contigs with an *N*_50_ of 92,830 bp, constituting a genome size of 4,349,822 bp. The assembled genome has a GC content of 54.5%. There are 107 contigs with an average coverage of 30×, and the average coverage across the genome is 46.7×. This genome was annotated using Rapid Annotation using Subsystem Technology (RAST), version 2.0 (7), which revealed the presence of 3,965 coding sequences and 77 RNA genes, with 59% of the coding sequences (CDS) covered by the subsystem in the RAST server. The Dickeya sp. 2B12 harbors putative pectate lyase and cellulase genes in its genome, which suggest the potential of Dickeya sp. 2B12 as a plant pathogen, a well-known role performed by other members of Dickeya spp. (1, 2). Interestingly, a pair of *luxI*/*luxR* homologues was also present in the genome in contig 11, suggesting the adaptation of a quorum-sensing system to regulate gene expression in this bacterium.

### Nucleotide sequence accession number.

This whole-genome shotgun project has been deposited at DDBJ/EMBL/GenBank under the accession no. JSYG00000000. The version described in this paper is the first version.

## References

[1] Toth IK, van der Wolf JM, Saddler G, Lojkowska E, Hélias V, Pirhonen M, Tsror L, Elphinstone JG 2011 *Dickeya* species: an emerging problem for potato production in Europe. Plant Pathol 60:385–399. doi:10.1111/j.1365-3059.2011.02427.x.

[2] Burkholder WD, McFadden LA, Dimock AW 1953 A bacterial blight of chrysanthemums. Phytopathology 43:522–526.

[3] Samson R, Legendre JB, Christen R, Fischer-Le Saux M, Achouak W, Gardan L 2005 Transfer of *Pectobacterium chrysanthemi* (Burkholder *et al.* 1953) Brenner *et al.* 1973 and *Brenneria paradisiaca* to the genus *Dickeya* gen. nov. as *Dickeya chrysanthemi* comb. nov. and *Dickeya paradisiaca* comb. nov. and delineation of four novel species, *Dickeya dadantii* sp. nov., *Dickeya dianthicola* sp. nov., *Dickeya dieffenbachiae* sp. nov. and *Dickeya zeae* sp. nov. Int J Syst Evol Microbiol 55:1415–1427. doi:10.1099/ijs.0.02791-0. 16014461

[4] Ma B, Hibbing ME, Kim HS, Reedy RM, Yedidia I, Breuer J, Breuer J, Glasner JD, Perna NT, Kelman A, Charkowski AO 2007 Host range and molecular phylogenies of the soft rot enterobacterial genera *Pectobacterium* and *Dickeya*. Phytopathology 97:1150–1163. doi:10.1094/PHYTO-97-9-1150. 18944180

[5] Dye DW, Bradbury JF, Goto M, Hayward AC, Lelliott RA, Schroth MN 1980 International standards for naming pathovars of phytopathogenic bacteria and a list of pathovar names and pathotype strains. Rev Plant Pathol 59:59153–59168.

[6] Parkinson N, DeVos P, Pirhonen M, Elphinstone J 2014 *Dickeya aquatica* sp. nov., isolated from waterways. Int J Syst Evol Microbiol 64:2264–2266. doi:10.1099/ijs.0.058693-0. 24719023

[7] Overbeek R, Olson R, Pusch GD, Olsen GJ, Davis JJ, Disz T, Edwards RA, Gerdes S, Parrello B, Shukla M, Vonstein V, Wattam AR, Xia F, Stevens R 2014 The SEED and the Rapid Annotation of microbial genomes using Subsystems Technology (RAST). Nucleic Acids Res 42:D206–D214. doi:10.1093/nar/gkt1226. 24293654 PMC3965101

